# Extrachromosomal circular DNA: Current status and future prospects

**DOI:** 10.7554/eLife.81412

**Published:** 2022-10-18

**Authors:** Yiheng Zhao, Linchan Yu, Shuchen Zhang, Xiangyu Su, Xiang Zhou

**Affiliations:** 1 Department of Cardiology, The Second Affiliated Hospital of Soochow University Suzhou China; Icahn School of Medicine at Mount Sinai United States; Icahn School of Medicine at Mount Sinai United States

**Keywords:** eccDNA, human diseases, biomedical research

## Abstract

Extrachromosomal circular DNA (eccDNA) is a double-stranded DNA molecule found in various organisms, including humans. In the past few decades, the research on eccDNA has mainly focused on cancers and their associated diseases. Advancements in modern omics technologies have reinvigorated research on eccDNA and shed light on the role of these molecules in a range of diseases and normal cell phenotypes. In this review, we first summarize the formation of eccDNA and its modes of action in eukaryotic cells. We then outline eccDNA as a disease biomarker and reveal its regulatory mechanism. We finally discuss the future prospects of eccDNA, including basic research and clinical application. Thus, with the deepening of understanding and exploration of eccDNAs, they hold great promise in future biomedical research and clinical translational application.

## Introduction

In 1965, Alix Bassel and Yasuo Hotta first reported their discovery of extrachromosomal circular DNA (eccDNA) in boar sperm and wheat embryos (Hotta and Bassel, 1965). Due to limited technology and a confusing system of classification and nomenclature, early research involving eccDNA was slow to start, which considerably hampered the characterization of the molecule. The rapid development of modern omics technologies and particularly whole-genome sequencing and bioinformatic analyses have greatly facilitated eccDNA characterization (Liao et al., 2020). This review will briefly introduce the current understanding of eccDNA formation and function and then focus on their potential therapeutic applications.

eccDNA has been identified in microorganisms, plants, model organisms, and human cells. eccDNAs are often categorized into small polydispersed circular DNA (spcDNA) (100–10,000 bp), episomes (submicroscopic size range), microDNA (200–3000 bp), telomeric circles (t-circles) (100–30,000 bp), double minutes (DMs) (100 kb3 Mb), and cancer-specific circular extrachromosomal DNA (ecDNA) (mega-base-pair amplified) (Noer et al., 2022; Zuo et al., 2021). Modern databases, including circle-map, Circle_finder, ecc_finder, CIDER-Seq, HolistIC, ECCsplorer, CircleBase, among others (Mann et al., 2022; Su et al., 2021; Zhang et al., 2021; Zhao et al., 2022), are constantly being established and updated to facilitate cataloging and classifying eccDNA from different species, and to characterize potential associations between human diseases and these molecules.

**Figure 1.**
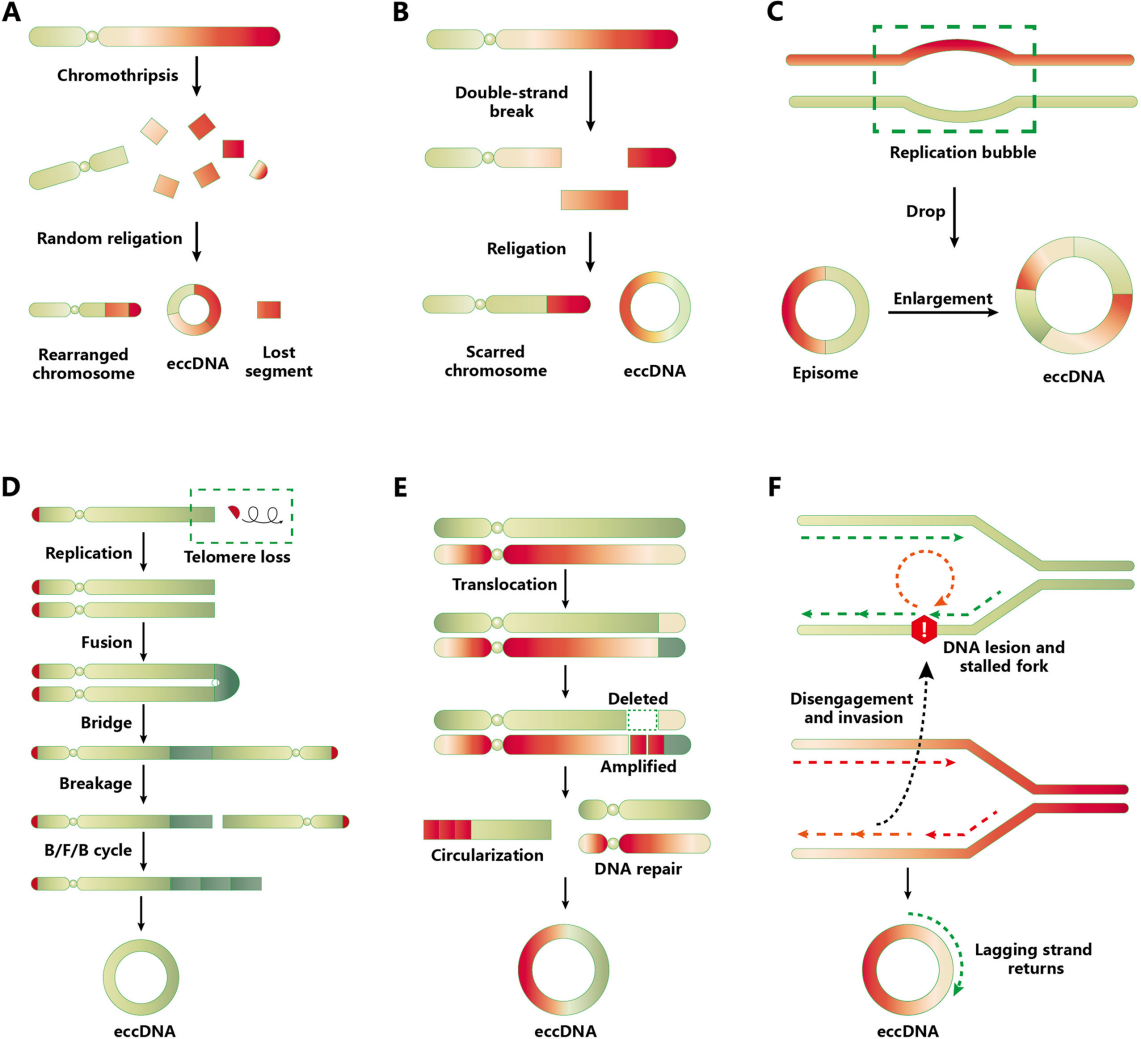
Models and pathways of extrachromosomal circular DNA (eccDNA) biogenesis. (**A**) Chromothripsis model. Chromothripsis causes DNA fragmentation through catastrophic chromosomal breakage. A portion of the fragments is reassembled randomly through DNA repair mechanisms, including homologous recombination and nonhomologous end-joining (NHEJ). During the repair process, eccDNAs are generated and their chromosomal segments are lost. (**B**) Mild DNA damage. Only one arm of a chromosome generates DNA fragment via DNA double-strand breakage, from which the putative eccDNA fragment is produced and also results in a scarred chromosome. The ligation of these DNA fragment contributes to eccDNA formation. (**C**) Episome model. A drop in the replication bubble can cause episome formation when errors occur in DNA replication. Episome replication or recombination leads to the formation of eccDNA. (**D**) Breakage–fusion–bridge (BFB) cycle. The loss of telomeres in the chromosome is the earliest event of the BFB cycle. After being replicated, the telomere-free chromosomes can fuse and form a dicentric anaphase bridge. The above cycle was repeated to prolong the telomere-free bridge, ultimately falling off and circularizing into eccDNA. (**E**) Translocation–excision–deletion–amplification model. When gene translocation occurs, the fragments adjacent to translocation positions can be amplified or deleted. Then eccDNA forms after the circularization of DNA fragments. (**F**) Fork stalling and template switching mechanism. When a DNA lesion exists following DNA bidirectional replication, the lagging strand anneals to the template strand of the adjacent replication fork through the microhomology mechanism to continue DNA synthesis. The process abovementioned may be repeated many times until the lagging strand returns to the original template.

A topic of interest is the biogenesis of eccDNA. Despite many studies being dedicated to answering this question (Cao et al., 2021; Ling et al., 2021), the complete mechanism remains unknown due to the variable structure and complex interactions of DNA molecules. Here, we summarize the different models and multiple pathways associated with eccDNA formation (Figure 1), including (1) chromothripsis, (2) mild DNA damage, (3) the episome model, (4) the breakage–fusion–bridge cycle, (5) the translocation–deletion–amplification model, and (6) fork stalling and template switching (FoSTeS). Microhomologous sequences are involved in the process of FoSTeS (Wu et al., 2022a), which may have several modes of action such as replication slippage, microdeletion, or microhomology-mediated end-joining pathway (Paulsen et al., 2018; Shibata et al., 2012; Zhang et al., 2009). However, direct evidence for these hypotheses is still lacking. A recent study (Bao et al., 2022) provided new evidence supporting the involvement of template switching in eccDNA formation. Bao et al. developed a novel computational algorithm termed Starfish, which can be used to classify the complex genomic rearrangements (CGRs) in cancer cells. These CGRs are grouped into six clusters according to the copy number and distribution of breakpoints. One of these clusters, which the authors named Signature 1, can be further divided into two signatures: ecDNA/DM and homogeneous staining regions (HSRs). ecDNA and HSR differ from each other in terms of their topology (circular and linear, respectively); hence, the article did not strictly differentiate them. Signature 1 breakpoints were highly enriched in head-on collision regions, which indicates that the conflicts between DNA replication and transcription may contribute to eccDNA formation in tumor tissues.

To date, studies have been primarily focused on the mechanism and function of eccDNA in cancer. Considering the large amount of research devoted to determining the function of eccDNA under different conditions, we classified the functions of eccDNA into the following categories: (1) oncogene amplification and transcription, (2) hijacking or mobile enhancers, (3) gene fusion and reintegration, (4) molecular sponge and transcriptional regulation (Paulsen et al., 2019; Yerlici et al., 2019), (5) telomere stability, and (6) cell signaling/communication (Yang et al., 2021a).

Overall, the biogenesis, forms, and functions of eccDNA are diverse and complex and many studies on eccDNA are not only limited to cancer, but also other diseases. Below, we summarize new findings relating to eccDNA formation and function and propose several directions for future investigations.

## eccDNA in human diseases

**Figure 2.**
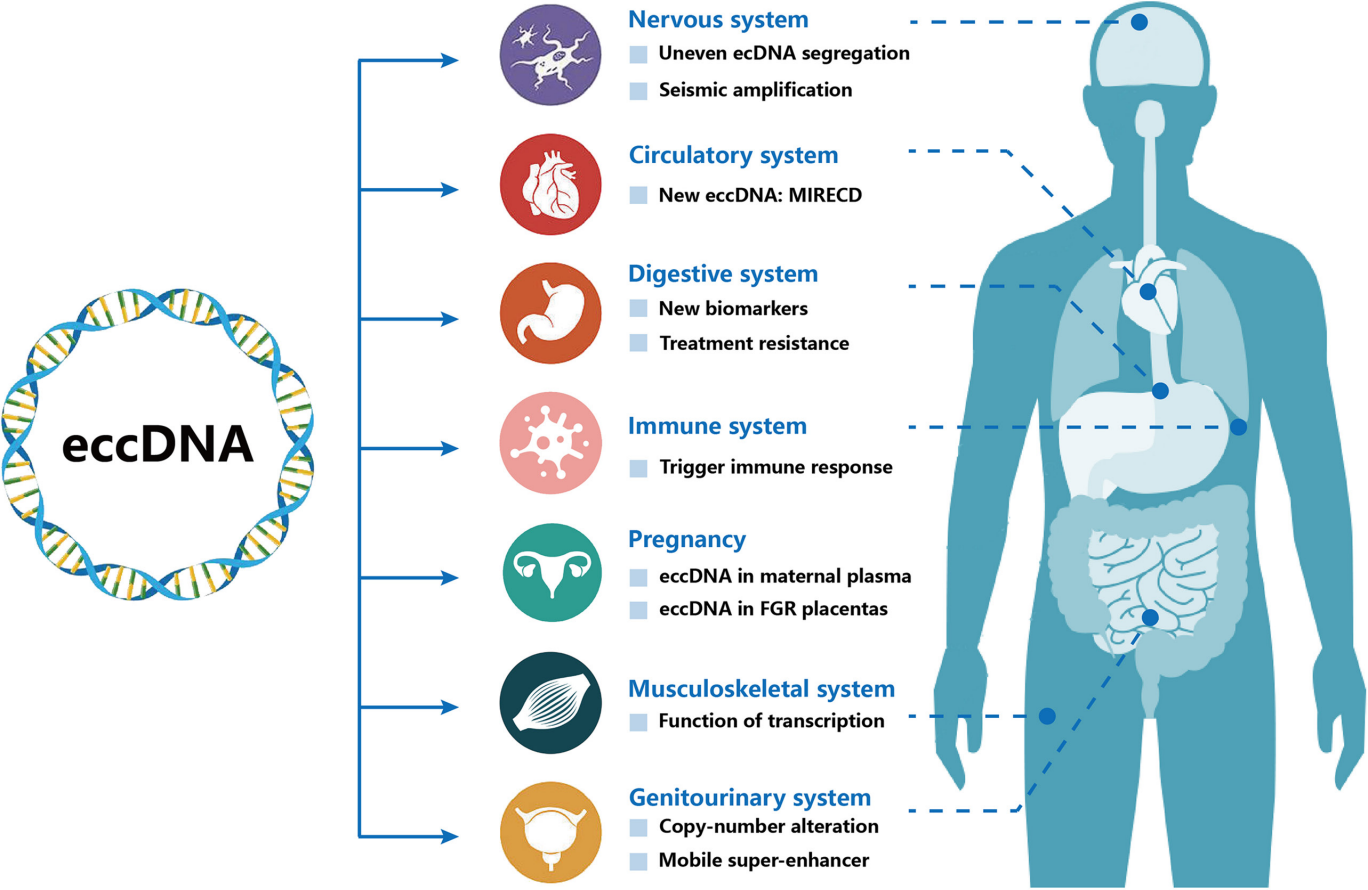
Extrachromosomal circular DNAs (eccDNAs) are associated with multiple human systems. Several eccDNAs have been identified in multiple human systems such as nervous system, circulatory system, digestive system, immune system, musculoskeletal system, and genitourinary system.

In recent years, numerous studies have demonstrated that eccDNAs may be involved in the development of various human diseases (Figure 2). In this review, we provide an overview of the role of eccDNAs as disease biomarkers and reveal their regulatory mechanisms in several diseases such as cancers, immunological disorders, and neurological and cardiovascular diseases (Table 1).

**Table 1.** Summary of extrachromosomal circular DNAs (eccDNAs) identified in various diseases.

Name	Disease	Function	Reference
eccDNA (EGFR)	Glioblastoma	Endogenous enhancer elements	Morton et al., 2019
ecDNA (ecEGFRx1, ecCCAT1, ecEGFR, and ecCCDC26)	Glioblastoma	Uneven segregation of ecDNA during mitosis	Yi et al., 2022
eccDNA (PDGFRA, CDK4)	Radiation-induced high-grade glioma	eccDNA-mediated amplification of oncogenes	DeSisto et al., 2021
eccDNA (TRPS1)	Breast cancer	TRPS1-driven genome deletions	Yang et al., 2021b
ecDNA (ecMYC)	Prostate cancer	Mobile transcriptional enhancers	Zhu et al., 2021
ecDNA/eccDNA (cyclin-E1, ERBB2, CDK12, EGFR, MYC)	Gastric cardia adenocarcinoma	Focal amplifications of oncogene prognostic molecular markers	Zhao et al., 2021
eccDNA (RAB3B)	Hypopharyngeal squamous cell carcinoma	Promote cisplatin resistance	Lin et al., 2022
eccDNA (MYCN, CDK4, MDM2)	Neuroblastoma	Seismic amplification model	Rosswog et al., 2021
eccDNA (entire genome)	Immune system	Trigger immune response	Wang et al., 2021
TTN^circle^	Musculoskeletal system	Function of transcription	Møller et al., 2018
MI-related eccDNA (MIRECD)	Myocardial infarction (MI)	MI prognosis prediction and risk stratification	Not yet published

### eccDNA in cancer

Numerous studies have suggested that eccDNAs are strongly associated with the oncogenesis or development and progression of tumors (Karami Fath et al., 2021; Ling et al., 2021; Wu et al., 2022b) and can function as a promising biomarker in diagnosing and the prognoses of these diseases. Many studies have focused on intracranial tumors, particularly glioblastoma (GBM) (Shiras and Mondal, 2021). In GBM, Morton et al. identified two enhancers located upstream of the *epidermal growth factor receptor* (EGFR) gene, which regulate EGFR gene amplification and subsequent cellular fitness (Morton et al., 2019). EGFR enhancers also increase extrachromosomal EGFR gene amplification; this change in amplification modifies chromatin topology and results in new contacts between the promoter and other enhancers within the loop domain. Their research further investigated whether a variation in enhancers on ecDNA can affect cell viability by modulating an oncogene. The conjecture was confirmed using the CRISPR interference screening approach. Tumor cells are rapidly proliferating malignant cells. It is also of interest to know how these molecules are transmitted to daughter cells and whether eccDNA is heritable. Many studies have shown the potential of uneven eccDNA segregation during mitosis, but direct evidence is still lacking (Verhaak et al., 2019). Recently, Yi et al. established a CRISPR-based DNA tracking system (ecTag) and confirmed the uneven segregation of eccDNA in GBM-derived neurosphere cells (Yi et al., 2022). It has also been reported that Cajal bodies and promyelocytic leukemia protein (PML) nuclear bodies contain hyperphosphorylated RNA polymerase II (RNAP II) (Gall et al., 1999; Kiesslich et al., 2002). The colocalization between Cajal/PML bodies and ecDNA confirmed that ecDNA hubs can function as sites of active transcription after mitosis (Yi et al., 2022). In addition to GBM, pediatric radiation-induced high-grade glioma (RIG) is also an intracranial tumor that commonly develops following cranial radiotherapy. DeSisto et al. identified two examples of chromothripsis-derived eccDNA when analyzing the focal genetic alterations of RIG that led to the upregulation of oncogenes such as platelet-derived *growth factor receptor alpha* (PDGFRA) and *cyclin-dependent kinase 4* (CDK4) (DeSisto et al., 2021).

Genitourinary tumors have also been the subject of many eccDNA studies, including breast and prostate cancer. During the exploration of breast cancer, Yang et al. unexpectedly found that the overexpression of the *transcriptional repressor gata binding 1* (TRPS1) gene could result in the deletion of genomic segments (Yang et al., 2021b). The authors then purified nonchromosomal DNA from normal human mammary epithelial cells (MCF-10A cells) that stably express TRPS1. High-throughput sequencing was then performed to determine the relationship between TRPS1 and gene copy loss. The sequencing data revealed that TRPS1 overexpression-associated genomic deletions were significantly enriched in eccDNA. Considering these molecules showed chromosomal-independent replication capacity, the authors hypothesized that eccDNA might function in oncogenesis. In addition, MCF-10A cells showed common and uniquely evolved sets of amplified copy-number alteration, thus suggesting an adaptation to antineoplastic drugs. Zhu et al. reported the chromatin connectivity networks of eccDNA in neurosphere and prostate cancer cells (Zhu et al., 2021). Zhu et al. performed chromatin immunoprecipitation (ChIP)-free ChIA-PET analysis in neurosphere cell lines and found eccDNAs can extensively bind to chromatin and function as transcriptional regulators involving the RNA polymerase II (RNAPII) complex. ChIP-sequencing (ChIP-seq) profiling of RNAPII binding and histone 3 lysine 27 acetylation (H3K27ac) modification confirmed an enhancer function for ecDNA sequences (Lovén et al., 2013; Whyte et al., 2013). Further analysis of chromosomal contact regions on ecDNA molecules has suggested that these loci bear superenhancer (SE)-like actions that can facilitate high target gene transcription (Hnisz et al., 2013). Zhu et al. designed synthetic eccDNA molecules and transfected them into ecDNA-negative prostate cancer (PC3) cells for further confirmation (Zhu et al., 2021). These authors validated the coaggregation of oncogenes by ecDNA SE in ecDNA-positive (ecMYC) cells, and they hypothesized that ecDNA-specific structures could promote tumorigenesis. No subsequent experiments have been performed to answer this hypothesis.

Research focusing on eccDNA-associated digestive system tumors has increased in recent years. Sun et al. performed Illumina high-throughput eccDNA sequencing to examine the distribution and function of eccDNAs in esophageal squamous cell carcinoma. Sun et al. identified five upregulated candidate eccDNAs, validated by PCR and Sanger sequencing (Sun et al., 2021). This study suggests that eccDNAs can function as biomarkers in cancer research. Zhao et al. explored the role of focal amplifications in gastric cardia adenocarcinoma (GCA) revealing an in-depth mechanism of eccDNA (Zhao et al., 2021). Zhao et al. first performed whole-genome sequencing and genome annotation to validate the existence of oncogene focal amplifications in GCA, including *cyclin-E1* (CCNE1), ERBB2, CDK12, EGFR, and MYC, among others. They then found that chromothripsis can lead to the progression of GCA through the focal amplification of oncogenes. They then compared the prognostic value of oncogene focal amplification in GCA and hypothesized that ERBB2 focal amplification might function as a good prognostic marker in GCA patients with the disease for longer than 2 years. Lin et al. showed that eccDNAs are also involved in treatment resistance (Lin et al., 2022). Using Illumina sequencing, Lin et al. defined the size distribution, gene location, and expression level of eccDNAs in hypopharyngeal squamous cell carcinoma. Subsequent analysis suggested RAB3B might be transcribed from eccDNA and could induce autophagy to exert cisplatin (DDP) resistance. The knockdown of RAB3B significantly enhanced the sensitivity of DDP and decreased their autophagy in DDP-resistant cells. However, Lin et al. did not provide experimental evidence to prove that transcriptional products were directly produced by eccDNA. Overall, these studies suggest eccDNA may have the potential to benefit the diagnosis, prognosis, and treatment of digestive system tumors.

### eccDNA and neurological diseases

DNA plasticity plays an important role in neurological diseases (Penndorf et al., 2018). Somatic mosaicism, including single-nucleotide variants, mobile element insertions, and structural changes in DNA, is ubiquitous in the human central nervous system. The current study mainly focused on the discovery and biogenesis of eccDNA in neural cells. Sekar et al. unintentionally discovered an eccDNA and a reciprocal chromosomal deletion in an adult neuron (Sekar et al., 2020). Following a prefrontal cortex dataset analysis, they found a deletion and duplication of chromosomal fragments on Chromosome Y in one of the study samples (Lodato et al., 2015). In addition, they highlighted that the deletion and duplication events occurred in an intergenic genomic region, and no microhomologies were observed between the breakpoints of the chromosome and the identified circular DNA. The authors speculated that the nonhomologous end-joining (NHEJ) DNA repair mechanism was involved in repairing DNA double-stranded breaks (DSBs). This hypothesis indicates that eccDNA originates from the NHEJ-mediated rejoining of the deleted genes after DSBs, though no further investigation was performed to confirm NHEJ is a mechanism for the generation of eccDNA molecules. Rosswog et al. proposed a new type of genomic amplification called seismic amplification in neuroblastoma (Rosswog et al., 2021). Seismic amplification is initiated by chromothripsis, and the DNA fragments realign randomly to form circular DNA structures. The stabilization of amplicons follows three distinct fates: (1) maintaining a circular structure as DMs, (2) integrating into a chromosome as homogeneously staining regions, or (3) forming neochromosomes. These mechanistic studies have deepened the understanding of eccDNA relating to neurological diseases and can provide new insights into the function of eccDNA.

### eccDNA in pregnancy and related disorders

Sin et al. identified the presence of eccDNA in the plasma of pregnant women (Sin et al., 2020). Circular DNA molecules were isolated from maternally derived plasma and linearized via MspI restriction digestion; next-generation sequencing was then performed to characterize the eccDNA species. The DNA fragments were classified into maternally and fetally derived eccDNAs characterized by single-nucleotide polymorphisms. Both groups exhibited two major peaks at 202 and 338 bp and showed 10 bp periodicity of eccDNA sizes. In another study, Sin et al. reported the methylation densities of both maternal and fetal molecules and found that the methylation of fetal eccDNA was significantly lower (p = 0.02, paired *t*-test) (Sin et al., 2021). The same study also showed that fetally derived eccDNAs were shorter than the maternally derived species and were rapidly reduced after birth. These characteristics have shown consistency between eccDNA and their linear counterparts in plasma. Genomic annotation also revealed that these molecules were enriched in 5′-untranslated regions (5′-UTRs), exonic regions, and CpG island regions, while Alu repeat regions had less enrichment (Sin et al., 2020). Another interesting finding of their study is the discovery of dual direct repeats in the head and tail regions of eccDNA molecules, which might function in the biogenesis of eccDNA through homologous recombination or microhomology-mediated end-joining mechanism (Mehanna et al., 2017; Møller et al., 2015; Paulsen et al., 2018). To investigate the potential mechanism of length variation of eccDNAs, the authors constructed knockout mouse models with deficiencies in deoxyribonuclease 1-like 3 (DNASE1L3) and found that plasma eccDNA in Dnase1l3^−/−^ mice exhibited larger size distributions than that in wild-type mice. eccDNAs in the maternal plasma were shorter in Dnase1l3^−/−^ mice pregnant with Dnase1l3^+/−^ fetuses than in the maternal plasma of Dnase1l3^−/−^ mice carrying Dnase1l3^−/−^ fetuses. This study demonstrates the biological association between plasma eccDNA and nuclease (Sin et al., 2022).

Such findings are not restricted to the plasma. According to a study by Yang et al., human placenta-derived eccDNAs also contained two peaks at 146 and 340 bp, and double-repeated trinucleotide sequences were equally present on both sides of these circular molecules (Yang et al., 2021a). In addition, their sequencing data revealed that the repertoires of eccDNAs in the placenta overlapped considerably with chromosomal regions, including gene regions, CpG islands, introns, and transposable elements (TEs). Yang et al. revealed that the length of eccDNAs corresponds to the wrapping length of DNA in nucleosomes, which indicates that eccDNAs are derived from nucleosomes (Dillon et al., 2015; Shibata et al., 2012). The distribution characteristics of eccDNAs, including GC content, genomic distribution, and repetitive sequences, were also consistent with those reported in previous studies on eccDNAs in humans and mice (Møller et al., 2018; Shibata et al., 2012; Zhu et al., 2017). However, important differences were identified between normal pregnancy and fetal growth restriction patients. A noticeable difference was the total number of eccDNAs, which was slightly higher than the control group (Yang et al., 2021a). Biological function analysis revealed apoptosis might play an important role in fetal growth restriction, which could, to some extent, relate to the shallow implantation of placental trophoblast cells (Paules et al., 2019). The same research also found that pathological states contributed to the elevation of eccDNAs in placentas, but whether these molecules were under the influence of gestational age requires further research.

### eccDNA in the immune system

The finding of immunostimulatory activity induced by eccDNAs through apoptosis was a breakthrough discovery regarding the role of eccDNA in the immune system (Wang et al., 2021). Wang et al. examined the genomic distributions of eccDNAs by using a novel purification method. The method consisted of three steps. First, DNA strands were extracted using a modified alkaline buffer at pH 11.8. Next, the restriction enzyme *Pac*I was used to linearize mitochondrial DNA (mtDNA) before the digestion of linear DNA by Plasmid-Safe ATP-dependent DNase. Finally, the silica column and silica beads were calibrated to further extract residual linear DNA in the solution. Mouse embryonic stem cells were selected as the main object of their research due to their genetic integrity. Although eccDNAs are widespread across the entire genome, unique characteristics such as their stochastic origins and fixed nucleosome sizes strongly suggest that the ligation of oligonucleosomal DNA fragments may be involved in the generation of these obscure molecules. There is substantial evidence demonstrating that these fragments are the feature of apoptosis (Marichal et al., 2011), and it is speculated that apoptotic DNA fragmentation (ADF) may participate in the formation of eccDNA (Wang et al., 2021). Follow-up experiments confirmed this speculation and revealed that the process was mediated through DNase γ (Shiokawa et al., 2002; Wang et al., 2021). Circularization research was performed on ADF using the CH12F3 mouse B-lymphocyte cell line and confirmed Lig3 was a vital enzyme for eccDNA generation (Lu et al., 2016; Wang et al., 2021). By using bone marrow-derived dendritic cells and bone marrow-derived macrophages, Wang et al. found that the circular structure of eccDNA and not the sequence stimulated the transcription of cytokine genes. The same study showed that the eccDNA molecules remained fully functional to induce an immune response through the coincubation of bone marrow-derived dendritic cells with the supernatant of apoptotic cells, which initiated a more robust cytokine activation than the cytokine inducer poly(dG:dC) (Wang et al., 2008). Gene ontology enrichment analysis and in vivo experiments on mouse lines suggested that eccDNA triggered innate immune responses through the Sting1 (stimulator of interferon genes) pathway. This research has provided an additional hypothesis that the genesis of cytokine storms in pathological conditions results from abnormal apoptosis (Moore and June, 2020; Wang et al., 2021).

The determination of how tumors escape the immune response is critical to understand the mechanisms of tumor progression. Wu et al. investigated the presence of ecDNA and its association with biomarkers of tumor immune evasion (Wu et al., 2022b). The authors found that the presence of eccDNA could result in a decreased immune cell infiltration status as measured by mRNA expression data. A gene set enrichment analysis revealed that major histocompatibility complex (classes I and II) associated genes were also significantly downregulated in tumors with ecDNA. The expression of immune inhibitory checkpoint genes such as PD-L1 and CTLA4 were also downregulated, maintaining tumor immunogenicity (Wu et al., 2022b). Consequently, these studies indicate that immune checkpoint inhibitor therapy is not a promising option for treating eccDNA-associated tumors.

### eccDNA in the musculoskeletal system

The study of Møller et al. provided new insights into the function of eccDNA in the musculoskeletal system. They identified junction transcripts from eccDNAs in muscle samples from healthy male volunteers. For instance, by comparing sequences between the transcripts of the TTN gene and the associated eccDNA sequences, they proved that its production resulted from TTN^circle^ and demonstrated that a fraction of eccDNAs residing inside the nucleus had transcription functions (Møller et al., 2018). A new study characterized plasma eccDNA in gouty arthritis and identified several eccDNA genes such as COL1A1 and EPB42, which were considered to be closely associated with hyperuricemia and gout (Pang et al., 2022). Several genes involved in inflammation or immune response were also identified by enrichment analysis. However, basic research on the mechanism by which eccDNA affects the musculoskeletal system is lacking, and an in-depth understanding of its role in musculoskeletal diseases is required.

### eccDNA in myocardial infarction

Our studies have shown that eccDNA may function as a biomarker for myocardial infarction (MI) prognosis (unpublished). We extracted plasma eccDNAs from the blood samples of patients with acute MI and performed circularization for in vitro reporting of cleavage effects by sequencing (CIRCLE-seq) to identify MI-related eccDNAs (MIRECDs). The CIRCLE-seq results were validated by qPCR analysis, and Sanger sequencing was used to validate the accurate sequence and the loop structure of the identified molecules. We identified a MIRECD and determined through gene annotation that it may function as an isoform-specific transcriptional enhancer by regulating different isoform usage of the Chromodomain Y Like gene. The Kaplan–Meier survival curves showed that high plasma MIRECD levels were associated with poor prognosis of acute MI. The incorporation of the expression of MIRECD into the prognostic model resulted in a better predictive value of the model. Our research confirmed that MIRECD could serve as a promising predictor for MI prognosis and risk stratification.

## eccDNA in other organisms

Apart from human cells, eccDNAs are widespread in eukaryotes, and most of these eccDNAs have wide-ranging bioactivities. To gain insights into the molecular mechanism of eccDNA, scientists have extensively used model organisms. Moller et al. developed the CIRCLE-seq method for the genome-wide detection of eccDNA. The CIRCLE-seq approach has demonstrated that eccDNAs are ubiquitous among yeasts (Møller et al., 2015). According to another study, the adaptability feature of copper resistance conferred by eccDNA to yeast proved crucial (Hull et al., 2019). Hull et al. revealed a correlation between accumulated copper and high levels of eccDNA containing a copper-resistance gene CUP1 (Hull et al., 2019). Yerlici et al. performed comprehensive investigations with *Oxytricha trifallax* and their research has captured rearrangement-specific circular DNA molecules across the genome, making *Oxytricha* a model system for studying nucleic acid topology (Yerlici et al., 2019). The functions of eccDNA in transcription and programmed genome rearrangement were also investigated. Studies on *Drosophila* are mainly focused on developing new mechanistic models such as the rolling circle replication of tandem genes (Cohen et al., 2005). Recently, Yang et al. reported that eccDNAs in *Drosophila* may serve as an additional source of TEs. They compared ratios of the eccDNA-TE counts between 30-day aged and 5-day young flies and concluded that eccDNAs as TEs accumulated during aging (Yang et al., 2022). *Xenopus* species have also been used to investigate the origin of eccDNA (Cohen and Segal, 2009). Moller et al. selected the domestic pigeon (*Columba livia domestica*) as a study organism and compared the variation of eccDNA from blood and breast muscle (Møller et al., 2020b). Their research revealed that the number of unique eccDNAs in a nonflying breed is higher than in homing pigeons, and the number of eccDNAs in pigeons was nine times higher than that in humans.

Plants also bear eccDNAs. For instance, eccDNA-associated glyphosate resistance was given significant attention in the field of plant science. Koo et al. hypothesized that the eccDNA in *Amaranthus palmeri* sporophytes could carry and amplify the 5-enolpyruvylshikimate-3-phosphate synthase (EPSPS) gene to acquire glyphosate resistance (Koo et al., 2018; Koo et al., 2021). In 2020, Molin et al. successively published three reports on glyphosate resistance and eccDNA replicons which involved multiple facets of regional distribution and genetic variation (Molin et al., 2020c; Molin et al., 2020a; Molin et al., 2020b). Gaines et al. found a massive eccDNA carrying various genes such as the EPSPS gene (Gaines et al., 2021). Diaz-Lara et al. proved the presence of eccDNA in Hop (*Humulus lupulus* L., family *Cannabaceae*) through rolling circle amplification (RCA) (Diaz-Lara et al., 2016), which indicated that RCA might be a useful tool to study eccDNA in higher plants. The relationship between TEs and eccDNA in plants has also been discussed in recent studies. eccDNAs derived from long terminal repeat retrotransposons (LTR-RTs) can be viewed as a mechanism that limits the number of new insertions of active retrotransposons (Lanciano et al., 2017). On the basis of this theory, Kwolek et al. described the LTR-RT landscape in the carrot genome and revealed through eccDNA sequencing that LTR-RTs are present and active in carrot callus cultures (Kwolek et al., 2022).

## New toolboxes for eccDNA research

High-throughput omics technologies are widely used in modern research, and the large datasets obtained from eccDNA sequencing must be analyzed using dedicated annotation platforms. Here, we describe the development of new tools for the detection and sequencing of eccDNA, many of which are based on short-read Illumina sequencing (Shibata et al., 2012). The CIRCLE-Seq procedure, in conjunction with the Circle-Map database (Prada-Luengo et al., 2019), is widely used in research on eccDNA characterization (Møller, 2020a). This methodology provides a sensitive and precise workflow to determine the short unaligned sequencing reads crossing circle junctions based on a probabilistic model. Compared to Circle-Map, ECCsplorer (Mann et al., 2022) has much broader applicability. Apart from the model organisms *Arabidopsis thaliana* and *Homo sapiens*, nonmodel organisms such as the crop sugar beet (*B. vulgaris*) can also be analyzed by ECCsplorer. The horizontal comparison of eccDNA candidates in humans showed ECCsplorer had a longer length distribution than Circle-Map (Mann et al., 2022). As no high-quality reference genome sequences are involved, this method shows high compatibility with assembly errors and is more appropriate for nonmodel organisms with low-coverage and short-read sequencing data. ATAC-seq is an assay for transposase-accessible chromatin sequencing and can identify incipient gene amplification in conjunction with the Circle_finder database, which is used to identify microDNA in human samples (Kumar et al., 2020). The advantages and disadvantages of the Circle_finder pipeline for analyzing ATAC-seq libraries deserve mention. Similar to the Starfish algorithm, Circle_finder cannot differentiate between eccDNA and chromosomal segmental tandem duplication (also known as HSR). However, the copy amplification length of eccDNA/segmental duplication is shorter than that of gene array hybridization. Comparison of ATAC-seq with whole-genome sequencing revealed that eccDNA/duplication events are somatic mosaics. Therefore, ATAC-seq can identify the incipient amplification of oncogenes in tumor cells and can serve as a useful approach to predict drug resistance.

Short-read technology has many strengths, including the high accuracy of sequencing reads and its accessibility to laboratories (Logsdon et al., 2020). However, limited read length is the biggest disadvantage of this method. This protocol cannot address large structural variants and repeat dense regions; therefore, long-read sequencing technologies were developed. Differing from the alignment-based algorithm in short-read technologies, genome assembly with long reads often relies on de novo assembly to determine the order of every base in a genome. CIDER-Seq (Mehta et al., 2020) is one of the few tools based on PacBio long reads that could obtain full-length sequences of eccDNAs. This technology can bridge gaps between the complex forms of genetic variation and short-read sequencing technology. However, the accuracy of this approach is subject to sequencing lengths. Moreover, the challenges to handle enormous datasets place new demands on computers. These limitations hamper the promotion of this research tool. CCDA-seq provides an approach different from ATAC-seq to reveal the chromatin status of integral eccDNA (Chen et al., 2021). Researchers have used methylase to label open chromatin and long-read nanopore sequencing to address the limitations of genome assemblies based on short-read sequencing. Another comprehensive tool known as ecc_finder (Zhang et al., 2021) appears to solve the read length problem by performing both short- and long-read sequencing. The high performance and short processing times make ecc_finder an optimal choice for processing large genomes.

An important feature of eccDNA is its ability to segregate into daughter cells unequally. SMOOTH-seq is developed for functional studies considering the importance of eccDNA detection at the single-cell level. This method is based on single-molecule, real-time sequencing technology (also known as PacBio sequencing), which enables accurate SV detection by taking advantage of long high-fidelity reads (Fan et al., 2021). An attractive feature of SMOOTH-seq is the clever use of commercialized Tn5 transposase with one embedded adaptor sequence. A single Tn5 adaptor sequence can avoid the loss of original DNA fragments, while low-concentrated Tn5 transposase can help to reduce the likelihood of self-looping. However, this innovation is a double-edged sword for eccDNA detection. A single sequencing read cannot recover the full-length sequence of long eccDNAs. Similar to many other methods, this method cannot differentiate between ecDNA and duplication events.

Different forms of eccDNA require specific approaches, and new methodologies are emerging to identify these molecules and characterize them. AmpliconArchitect (AA) and AmpliconReconstructor (AR) are extensively used to define ecDNA in tumors (Deshpande et al., 2019; Luebeck et al., 2020). Reconstructing possible ecDNA and other focal amplicon structures from short-read data is one of the great advantages of AA. However, the sequencing technology depends on extracting paths and cycles from the breakpoint graph, and it can be used only as a template for guiding assembly of longer, single-molecule reads instead of the accurate reconstruction of ecDNA molecules. The subsequent AR integrates optical mapping of long DNA fragments (>150 kb) with next-generation sequencing to realize the reconstruction of fine- and large-scale ecDNA structure. HolistIC (Hayes et al., 2021) is a tool for the discovery of DMs. The HolistIC algorithm relies on whole-genome shotgun sequencing data and the prediction of overlapping amplicon coordinates. These features are advantageous for revealing the associations between the amplicons and eccDNA. Sequencing techniques are continuously evolving for enabling sequencing accuracy. The majority of mtDNA can be successfully removed by the endonucleases *Mss*I and *Pac*I according to the existing methods. However, it could also affect the large-sized eccDNAs, especially those containing the restriction enzyme target sites. Feng et al. developed a method that can selectively remove mtDNA from total circular DNA under the guidance of the CRISPR/Cas9 system with one single-guide RNA (sgRNA) or two sgRNAs. The method could also be applied to any eukaryotic species, indicating that this technology is expected to play a role in protecting large DNA circles from endonucleases (Feng et al., 2022). As the use of CRISPR/Cas9 continues to expand in detecting variants, the tracing of eccDNA may have more than one approach. In addition to ecTag mentioned previously, Lyu et al. invented a new DNA live-imaging system called CRISPR-mediated fluorescence in situ hybridization amplifier (CRISPR FISHer). The CRISPR FISHer is composed of nuclease-deactivated dCas9 and sgRNA with two PP7 RNA aptamers (sgRNA-2 × PP7) which could recruit a fusion protein consisting of the PP7 coat protein (PCP), green fluorescent protein (GFP), and a T4 fibritin trimeric motif foldon 14–16 (termed foldon-GFP-PCP). The eccDNA junction sequences were selected as CRISPR FISHer targeted sites to detect and track the movement of eccDNA (Lyu et al., 2022).

Machine learning is also a powerful tool for downstream bioinformatics analyses. To thoroughly investigate the interactions between different eccDNAs, it is necessary to build a platform that can integrate the large datasets generated by eccDNA studies. CircleBase (Zhao et al., 2022), a recently developed platform summarizes the functional mechanisms of eccDNA and facilitates the further exploration of genome diversity and variability.

## Conclusions and perspective

**Figure 3.**
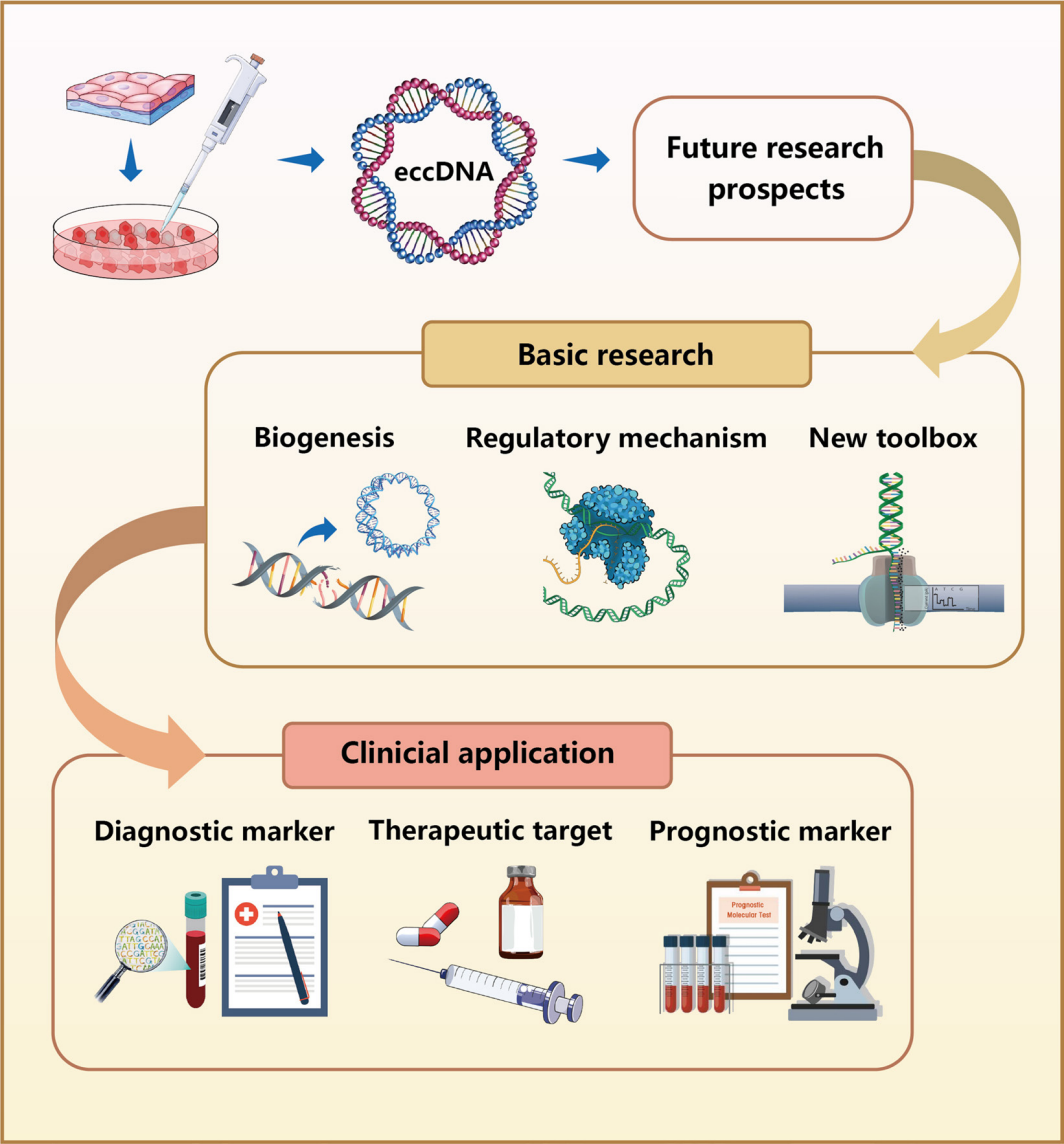
Future research prospects for eccDNAs. In basic research, the biogenesis of eccDNAs remains unclear, although numerous mechanistic models have been proposed. Further studies are required to investigate the regulatory mechanisms of eccDNAs in the occurrence and development of various diseases. Because of the limited tools currently available for analyzing eccDNAs, the development of new research methods is imperative. In clinical application, eccDNAs can be used as diagnostic and prognostic biomarkers because of their stable presence in human plasma. Moreover, eccDNAs are expected to serve as therapeutic targets for treating various diseases.

Collectively, eccDNAs are broadly present in eukaryotes. Despite the important advances recently made in this field, eccDNA research is still in its infancy with many aspects that must be improved upon (Figure 3). First, previous studies are mainly focused on tumor cells, such as the origin and composition of eccDNA in tumor cells. Very few researchers have thoroughly investigated the function of eccDNA in other diseases, but increasing evidence indicates that eccDNA plays an essential role in the development of multiple diseases. Second, the mechanism of action of eccDNA is still poorly understood because of methodological limitations. New targets and screening approaches in eccDNA discovery are scarce and limited to a single-type approach. These factors make it difficult to determine whether the identified molecules are functional. Last but not least, a wide range of issues involving the definition and classification of eccDNA are emerging with further in-depth studies. For instance, the concept of DMs seems to overlap with that of ecDNA, and the subclassification proposed based on different perspectives such as biogenesis and size difference adds to the confusion.

Although many emerging research questions related to the function and mechanism of eccDNA are emerging following the use of eccDNA sequencing and analysis, these issues will certainly provide an impetus for conducting future studies in this direction. For example, study topics should not be restricted to tumor cells, and eccDNAs from other pathological tissues and peripheral blood samples can be considered as biomarkers of tissue-specific diseases. Investigations in animals and plants suggest eccDNA participates in the evolution of different species (Arrey et al., 2022; Gaines et al., 2021). Easy access to cells and tissues would make it more convenient to generate reliable results, which can be mined to generate new hypotheses and theoretical guidance for further research. Additionally, basic research in new fields requires the development of new tools, especially when high-throughput technologies are the standard approach. Although experimental techniques are being rapidly developed, the current methods have limitations such as read length or complex protocols (Mehta et al., 2020). The sequence similarity between eccDNA and TEs remains a topic of interest, but the research focus is shifting toward the function and application of these molecules. Much of the functional studies available involve high-throughput sequencing analysis, which lacks direct experimental evidence to bolster their conclusions. The primary issue is how to synthesize specific eccDNAs and import them into cells in vitro. However, to date, very few studies have investigated the feasibility of this approach. It is also impossible to classify the whole range of eccDNAs by determining the origin or length alone. The tremendous variation among eccDNAs indicates that their defining characteristics should be refined to facilitate future studies.

In addition to research on eccDNA mechanisms and characteristics, eccDNA may also have excellent prospects for clinical application. Firstly, available data from studies suggest eccDNA may act as an alternative biomarker to increase the diagnostic accuracy of rare diseases (Aghamir et al., 2020; Ain et al., 2020). Combining different biomarkers could allow for a more precise clinical assessment of a patient’s condition. Secondly, eccDNA can serve as drug targets and antitargets for disease therapy. As detailed in this review, oncogene amplification and drug resistance result from eccDNA discovered in experimental and clinical studies. Therefore, it is reasonable to believe that eccDNA intervention can regulate gene expression or enhance drug sensitivity to improve treatments. Thirdly, the function of eccDNA in the prognosis of diseases is also a promising research direction. Different levels of plasma eccDNA can be used for risk stratification of disease and subsequent assessment of treatment effects. In conclusion, eccDNA holds enormous promise in future disease research.
